# The Linear Relationship Model with LASSO for Studying Stock Networks

**DOI:** 10.3390/e24060808

**Published:** 2022-06-09

**Authors:** Muzi Chen, Hongjiong Tian, Boyao Wu, Tianhai Tian

**Affiliations:** 1 School of Management Science and Engineering, Central University of Finance and Economics, Beijing 102206, China; zizizhuzhu0320@163.com; 2 Department of Mathematics, Shanghai Normal University, Shanghai 200234, China; hjtian@shnu.edu.cn; 3 School of Banking and Finance, University of International Business and Economics, Beijing 100029, China; 4 School of Mathematics, Monash University, Clayton, VIC 3800, Australia

**Keywords:** stock relationship network, linear relationship model, LASSO, negative correlation

## Abstract

The correlation-based network is a powerful tool to reveal the influential mechanisms and relations in stock markets. However, current methods for developing network models are dominantly based on the pairwise relationship of positive correlations. This work proposes a new approach for developing stock relationship networks by using the linear relationship model with LASSO to explore negative correlations under a systemic framework. The developed model not only preserves positive links with statistical significance but also includes link directions and negative correlations. We also introduce blends cliques with the balance theory to investigate the consistency properties of the developed networks. The ASX 200 stock data with 194 stocks are applied to evaluate the effectiveness of our proposed method. Results suggest that the developed networks not only are highly consistent with the correlation coefficient in terms of positive or negative correlations but also provide influence directions in stock markets.

## 1. Introduction

The world is experiencing revolutions in a wide range of areas in the era of Big Data. Although substantial progress has been made recently towards the statistical analysis and machine-learning studies of big data, there are still significant challenges for further exploiting valuable information from the big datasets. The network theory, which has been shown as a powerful tool [1,2,3], can capture critical features of systems, filter out redundant information, and provide insightful solutions to data problems in finance, economics, biology, social science, engineering and physical sciences [4,5,6,7,8,9,10,11].

One of the key questions in financial network analysis is how to describe underlying interactions in the stock market network. The original work employs the minimum spanning tree (MST) to extract the hierarchical structure of stocks traded on the US stock exchanges [12]. Since then, two significant issues have been widely discussed to reveal synchronous relationships in stock networks: how to quantify the relationship between different stocks and how to generate sparse stock networks based on the relationship measures.

Regarding the relationship measure, the Pearson correlation is the predominantly used method to calculate the similarities between stock pairs [12,13,14]. Such co-movement patterns are further described by the co-integration tests [15] and partial correlations [16,17,18]. In addition, the causality is utilized to characterize the “before-after” relationships in systems [19,20]. We recently proposed to use mutual information to measure the nonlinear dependencies between stocks [21,22]. The information theory has been used recently to study stock relationship networks and other related financial problems [23,24,25,26,27,28,29,30,31,32]. It is important to emphasize that the complexity of stock market systems may lead to a number of interactive patterns among stocks, and hence different measurements are needed to produce financial networks.

Regarding the construction of stock networks, the MST, asset graph (or the threshold networks) [33,34] and planar maximum filtered graph (PMFG) [13] are the three major methods that have been widely applied to analyse a series of important issues in stock markets [35]. For example, as an essentially growing market, the Chinese stock market exhibits much stronger correlations than the markets in developed countries [36]. Another important topic is the performance of stock markets during the financial crisis [37]. Studies show that stock networks have a more concentrated topological structure during financial crises than other time periods [38]. Recently we developed a multi-likelihood method for developing stock relationship networks using multiple threshold values [39].

A widely used approach in the correlation-based methods is to transfer the correlation coefficient $\rho_{ij}$ for stock *i* and stock *j* into the distance using $d_{ij}=\sqrt{2(1-\rho_{ij})}$. Then links are added into networks for those with small distances (large positive coefficients). Given that negative correlations always have larger distances than positive ones and the generated network is sparse, negative relations are excluded in the construction in practice. In addition, the threshold methods typically use $\rho_{ij}>c_{0}$ as the criterion to filter out redundant information [34], which also excludes negative correlations from discussions. Although the negative correlation is a key issue in other networks such as the genetic regulatory networks [40,41], this topic is less addressed in the three methods discussed above. In addition, existing methods mainly rely on bilateral relationships between paired stocks, which cannot reflect the fact that many other stocks may also influence a given stock simultaneously. Furthermore, correlation-based methods suffer from the potential vulnerable clustering structures because not all pairs with high Pearson correlations are corresponding to reliable connections in reality [14].

This work proposes a novel method to develop correlation-based networks by employing the linear relationship model with LASSO (LRML). To find the influential factors, we use the LASSO inference method with the adjustable penalty to tune the sparseness of networks and search for reliable relations whose corresponding coefficients are significant statistically. The relative methodologies on LASSO have been well studied [42,43,44,45], and applied into forecasting problems in finance [46,47,48] as well as building genetic networks [49]. By contrast, our work aims at developing a unique model that is consistent with the correlation methods to study the synchronous behaviour in stock markets. Compared with the existing methods dominantly based on pairwise relationships, the proposed method can develop stock correlation networks from the systematic view involving both positive and negative connections. Empirical studies on the ASX 200 stock market demonstrate that our method maintains the main topological structure from traditional correlation-based approaches. In addition, the developed networks include directions, signs and statistical significance in the same framework. Consequently, the proposed LRML provides a systematic regulatory and statistic view to make better use of data and further understand financial systems.

## 2. Methods

### 2.1. Linear Relationship Model with LASSO (LRML)

In this work we introduce the following multivariate linear model to describe the synchronous relations between stocks. The price of a stock is linearly modelled by the prices of other stocks at the same time, given by

(1) $$x_{it}=\alpha_{i}+\sum_{j=1,j\neq i}^{N}\beta_{ij}x_{jt}+\varepsilon_{it},\;i=1,\ldots ,N,$$

where $x_{it}$ is the price of the *i*th stock at time *t*, $\alpha_{i}$ the individual effect of stock *i*, $\beta_{ij}$ the impact of stock *j* on stock *i*, and $\varepsilon_{it}$ the error term. Similar to the regulations in genetic networks, it is assumed that a positive (negative or zero) value of $\beta_{ij}$ reflects positively (negatively or no) influences from stock *j* to stock *i*.

Unlike the widely used regression models or predictive models [44,45,46,47,48,49], the values of the left-hand side in (1) are the stock prices at time *t* rather than at time (t+1) in order to be consistent with the existing approaches using Pearson correlations. Thus, Equation (1) includes the correlation-based methods if only one stock *j* has the influence on stock *i*, given by

$$x_{it}=\alpha_{i}+\beta_{ij}x_{jt}+\varepsilon_{it}.$$

In this case, coefficient $\beta_{ij}$ has the same sign as its corresponding correlation coefficient $\rho_{ij}$ between stocks *i* and *j*.

There are a number of established methods for inferring the values of $\alpha_{i}$ and $\beta_{ij}$. The challenge is how to build networks with the similar sparsity as those constructed by current approaches. In this paper, the unknown coefficients are estimated by the LASSO method in [50,51]:

(2) $${\hat{\beta }}_{i}=\underset{\beta_{i}}{\mathrm{argmin}}\left[\frac{1}{2}\sum_{t=1}^{T}(x_{it}-\alpha_{i}-\sum_{j=1,j\neq i}^{N}\beta_{ij}x_{jt}{)}^{2}+\lambda \sum_{j=1,j\neq i}^{N}\left|\beta_{ij}\right|\right],$$

where λ is the penalty factor controlling the number of none-zero coefficients. Generally, a larger value of λ leads to more zero parameters in Equation (1) and fewer links in the created network. In this work, we do not pursue the optimal solution of λ but treat this penalty factor as a given parameter for adjusting the density of developed networks.

For the stock network defined by Equation (1), we use A to denote the adjacency matrix whose element $a_{ij}$ is given by

(3) $$a_{ij}=\left\{\begin{array}{cc} \frac{{\hat{\beta }}_{ji}}{|{\hat{\beta }}_{ji}|}, & |{\hat{\beta }}_{ji}|\neq 0;\\ 0, & |{\hat{\beta }}_{ji}|=0. \end{array}\right.$$

Equation (3) defines a directed and signed network. The link from stock *j* to stock *i* exists as long as coefficient $\beta_{ij}$ is non-zero, and this edge shares the same sign with its corresponding coefficient. Since this adjacency matrix may be asymmetric, it will be difficult to apply the balance theory to the signed graphs and conduct comparison studies with the existing correlation-based methods. Thus, the symmetric adjacency matrix B with elements $b_{ij}$ is defined as:

(4) $$b_{ij}=b_{ji}=\left\{\begin{array}{cc} \frac{{\hat{\beta }}_{ij}+{\hat{\beta }}_{ji}}{|{\hat{\beta }}_{ij}\left|+\right|{\hat{\beta }}_{ji}|}, & |{\hat{\beta }}_{ij}\left|+\right|{\hat{\beta }}_{ji}|\neq 0;\\ 0, & |{\hat{\beta }}_{ij}\left|+\right|{\hat{\beta }}_{ji}|=0. \end{array}\right.$$

Thus, matrix B simplifies the structure of matrix A such that an undirected link in B exists if there is at least one directed edge between the considered stock pair in A. However, no undirected link exists if the two directed edges between two stocks have the opposite signs.

There are three special types of links in the our constructed networks: homogeneous links (i.e., $a_{ij}=a_{ji}$ in matrix A), heterogeneous links (i.e., $a_{ij}=-a_{ji}$), and singular links that are proposed for the newly appeared links or links changing their signs when λ grows. The singular links include four cases: (1) the sign of $\beta_{ij}$ changing from positive to negative, (2) from negative to positive, (3) from zero to positive, and (4) from zero to negative. Both heterogeneous links and singular links are important since either differences in signs or changes in links will cause confusion about the determination of corresponding connections in networks.

### 2.2. Network Density

We employ the network density to evaluate the sparseness of developed networks and the influence from three different types of links (i.e., homogeneous, heterogeneous and singular links) on the networking structure. This concept is defined by the proportion of links in the given network to those of the fully connected network, namely

(5) $$\mathrm{Density}=\frac{\sum_{i,j=1,i\neq j}^{N}|a_{ij}|}{N(N-1)},$$

where *N* is the stock number and $a_{ij}$ is defined in Equation (3). Inference results later suggest that the penalty factor λ in Equation (2) largely dominates the density of the developed networks. A larger value of λ leads to a sparser network with fewer heterogeneous and singular links.

### 2.3. Topological Properties of Signed Networks

The balance theory, widely used in signed graphs, derives from the famous motto “enemies’ enemies are my friends” [52]. A subgraph is balanced if it has an even number of negative links, implying a harmonious and stable situation where a graph can be divided into several subgraphs such that positive and negative edges are highly possible to exist within subgraphs and between subgraphs, respectively. Since networks created by the LRML are bilateral, we neglect directions of all edges and use the symmetric adjacency matrix B in Equation (4) when considering the balance theory.

According to graph theories, a clique in undirected graphs is a maximal complete subgraph of three or more nodes [52]. However, it would be difficult to extend this definition from undirected graphs to directed graphs, because all bilateral relations are required for all stock pairs in the directed subgraph and bilateral relations between two stocks should have the same signs. In this paper, we use a weaker definition for directed and signed networks to discuss cliques and balance theory. A subset in directed networks is defined as a clique if there is at least one link between any node pairs in the subset. We also propose a concept called “intensity” to measure the sparsity of concerned cliques. For a clique with *k* nodes, the minimal and maximal directed links are k(k−1)/2 and k(k−1), respectively. Thus, the intensity of a k-clique with *L* directed links is defined as

(6) $$\mathrm{InT}\left(k,L\right)=\frac{L-k(k-1)/2}{k(k-1)/2}=\frac{2L}{k(k-1)}-1,$$

Thus 0≤InT(k,L)≤1. A larger value of intensity implies the closer and tighter structure in the given clique.

## 3. Results

### 3.1. Stock Price Data and Pearson Correlations

We use the Australian ASX 200 daily trading data over the time period from 1 July 2016 to 30 June 2017 (253 trading days) to evaluate the proposed LRML model. As the benchmark for Australian equity performance, the ASX 200 consists of the 200 largest stocks and accounts for about 82% of the Australian share market capitalisation. This index is a capitalisation weighted and float-adjusted stock market index listed on the Australian Securities Exchange. Data are downloaded from Yahoo Finance whose website address is https://au.finance.yahoo.com/ (accessed on 1 December 2017). Six stocks (MTR, BTT, DHG, CLW, VVR and WFD) are removed from our analysis due to the incomplete data over the concerned trading days.

**Figure 1:**
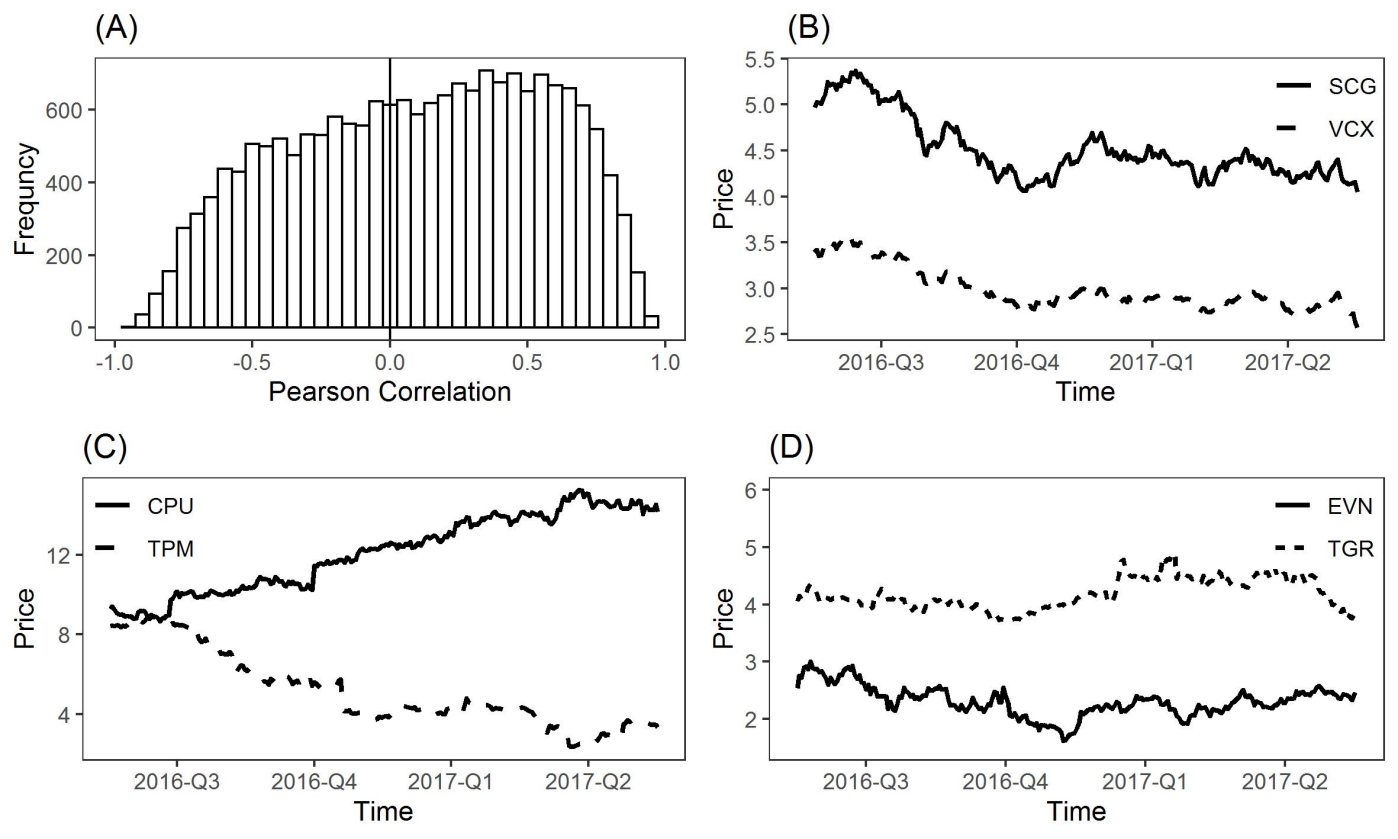
Importance of negative correlation coefficients. (**A**) Histogram of all Pearson correlation coefficients among ASX 200 stock pairs. (**B**) Example of a stock pair with the positive correlation coefficient (+0.97) (Solid-line: SCG, Scentre Group; Dash-line: VCX, Vicinity Centres). (**C**) Example of a stock pair with the negative correlation coefficient (−0.93) (Solid-line: CPU, Computer share; Dash-line: TPM, TPG Telecom). (**D**) Example of a stock pair with the small absolute value of the correlation coefficient ($-1.6\times 10^{-5}$) (Solid-line: EVN, Evolution Mining Limited; Dash-line: TGR, Tassal Group Limited). Here, “2016-Q3” in x-axis means the third quarter in 2016.

To demonstrate the importance of negative correlations, Figure 1A presents the histogram of Pearson correlation coefficients for all stock pairs. Among the total of ∼18,600 correlation coefficients, 41.6% (i.e., 7788) of them are negative. In particular, there are 688 and 200 correlation coefficients whose values are greater than 0.8 and less than −0.8, respectively. Since most links with high positive correlations are likely to be added into the correlation-based network (e.g., MST, PMFG, or asset graphs), we have sufficient reasons to equally consider those negative correlations with high absolute values. These negative correlations may also play an important role in developing stock networks. In addition, the numbers of correlation coefficients with values in [0.6,0.8], [0.4,0.6] and [0.2,0.4] (or [−0.8,−0.6], [−0.6,−0.4], and [−0.4,−0.2]) are 2387, 2672 and 2718 (or 1247, 1910, and 2089), respectively. Since negative correlations widely exist in different ranges as positive ones, networks involving both positive and negative relations can make better use of the data.

Figure 1 provides three examples of the positive relationship between SCG (Scentre Group) and VCX (Vicinity Centres) with a high value of correlation coefficient +0.97, negative relationship between CPU (Computershare) and TPM (TPG Telecom) with −0.93 and no correlated relationship between EVN (Evolution Mining Limited) and TGR (Tassal Group Limited) with a small value of the correlation coefficient $-1.6\times 10^{-5}$. The high correlation between SCG and VCX is the result that both companies belong to the real estate sector. In addition, TGR and EVN are in the consumer staples and material sectors, respectively, and these sectors have different business activities. However, CPU and TPM are in the information technology and telecommunication services sectors, respectively. The changes in share prices may reflect the trend of business activities in these two sectors.

### 3.2. The Influence of Penalty Factor

**Figure 2:**
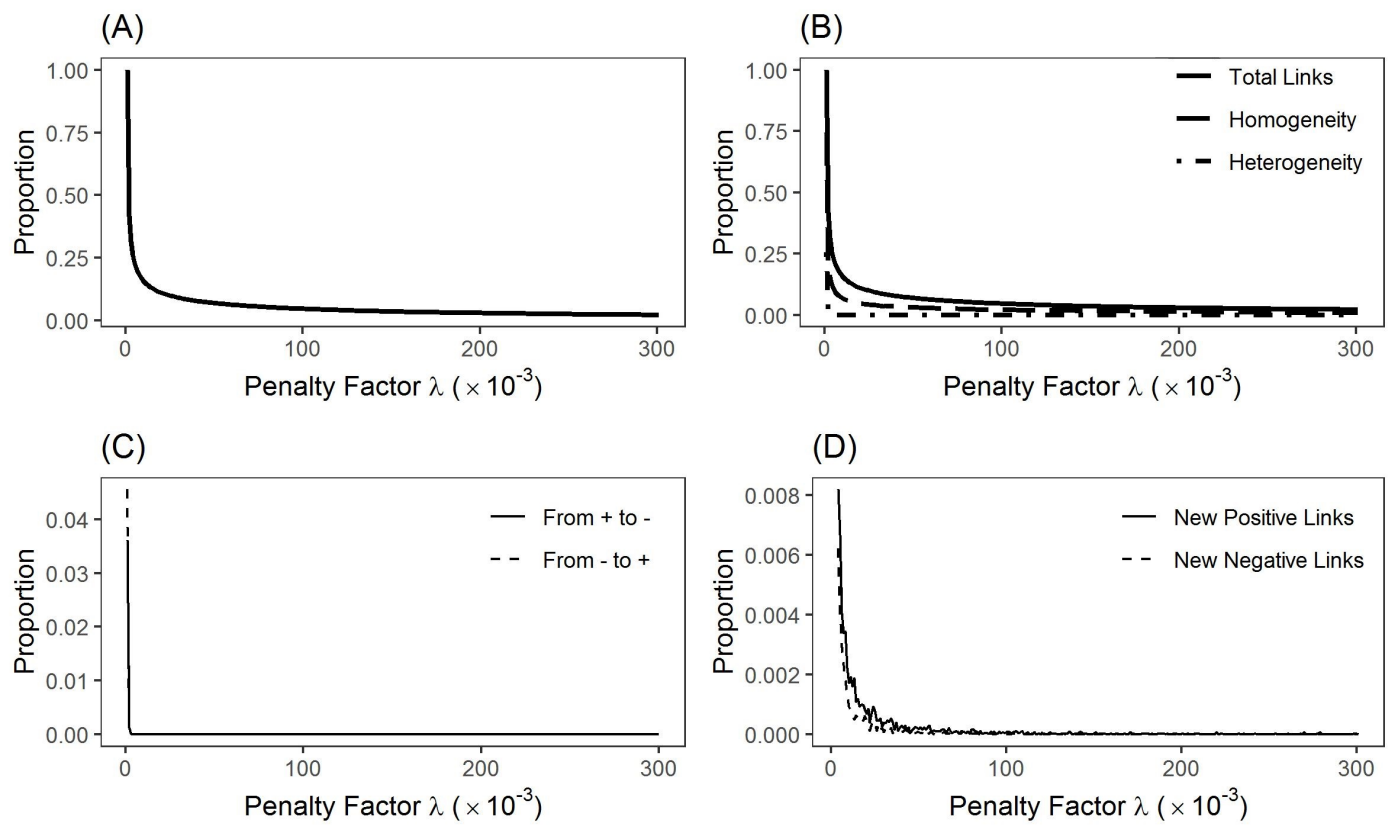
Influence of penalty factors on the network structure. (**A**). Network densities determined by different values of λ. (**B**). Proportions of homogeneous links and heterogeneous links determined by different values of λ (Solid-line: proportions of all links; Dash-line: proportions of homogeneous links; Dash-dot line: proportions of heterogeneous links). (**C**). Proportions of singular links with the changing signs determined by different values of λ (Solid-line: proportions of links changing from positive correlations to negative ones; Dash-line: proportions of links changing from negative correlations to positive ones). (**D**). Proportions for the appearance of new positive and negative links determined by different values of λ (Solid-line: proportions of links appearing as new positive correlations; Dash-line: proportions of links appearing as new negative correlations).

We next evaluate the impact of the penalty factor on the network structure. Figure 2A presents the densities of the generated networks by using different values of the penalty factor. As the penalty factor λ rises from zero, the network density falls sharply at the beginning. However, after the density is below 0.2, the further increase of the penalty factor only decreases the density moderately. Figure 2B shows how homogeneous links (i.e., $a_{ij}=a_{ji}$) and heterogeneous links (i.e., $a_{ij}=-a_{ji}$) change as λ increases. For comparison, the network density in Figure 2A is also presented in Figure 2B. It shows that homogeneous links always exist in networks created by different values of λ, while heterogeneous links disappear when the penalty factor is larger than a small value (λ>0.02). Regarding singular links, Figure 2C shows the percentages of singular links with the changing signs, and Figure 2D provides the percentages of singular links for the appearance of new links. Similar to heterogeneous links, the number of singular links is less than 10 when the penalty factor is larger than a small value (λ>0.057), which is negligible in the produced network. Therefore, to develop a sparse network with adequate large values of penalty factor (e.g., λ>0.05), neither the heterogeneous nor singular link is an issue in constructing stock networks.

### 3.3. Consistency of Pearson Correlation and LRML

**Figure 3:**
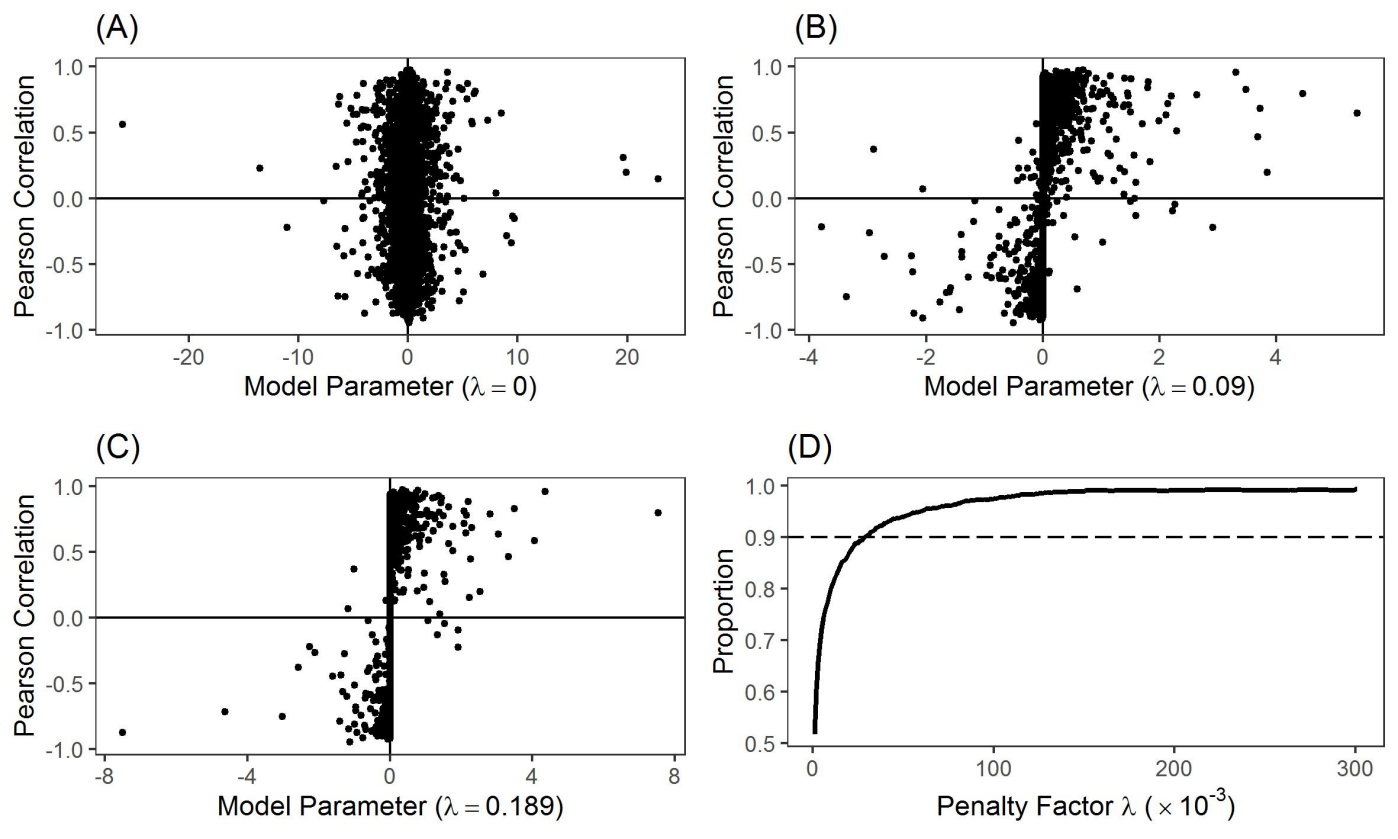
Relationships between model coefficients and Pearson correlation coefficients. (**A**) Network with full links using λ=0. (**B**) Network determined by λ=0.09. (**C**) Network determined by λ=0.189. (**D**) Percentages of dots in the first and third quadrant for the networks determined by different penalty values.

A key question in our study is whether links in the LRML network have consistent signs with their corresponding Pearson correlations. To answer this question, we plot the Pearson correlation coefficients $\rho_{ij}$ against the model coefficients $({\hat{\beta }}_{ij}+{\hat{\beta }}_{ji})/2$ in Figure 3 for three networks determined by penalty factors λ=0, 0.09 and 0.189. Dots in the first and third quadrants represent stock pairs whose signs of Pearson correlation coefficients are the same as those of model links. However, dots in the second and fourth quadrants represent stock pairs whose signs of Pearson correlation coefficients are opposite to those of model links. Here we consider the average coefficient $({\hat{\beta }}_{ij}+{\hat{\beta }}_{ji})/2$ in order to compare these two parameters with the single value of correlation coefficient $\rho_{ij}$. Since Figure 2B shows that the heterogeneous links nearly disappear in the developed networks in Figure 3B,C, there would not be any cancellations between the values of ${\hat{\beta }}_{ij}$ and ${\hat{\beta }}_{ji}$.

For the network with full links (λ=0), Figure 3A displays there are nearly a half of stock pairs (i.e., ∼48.3%) located in the second and fourth quadrants. Figure 3B,C indicate that the rise of penalty factor substantially decreases the conflicts between the Pearson correlation coefficients and our model coefficients. To be specific, without considering the directions of links, 30.8% and 20.8% generated links appear in the first and third quadrant of Figure 3A, 71.0% and 26.2% in Figure 3B and 76.1% and 22.9% in Figure 3C, respectively, which also shows the importance of negative influence from stocks. When the network becomes sparser by using a larger value of λ, the model coefficients tend to have the same signs as the corresponding Pearson correlation coefficients. Figure 3D gives the percentages of dots in the first and third quadrant for the networks determined by different penalty values. Starting from ∼51.7% without any penalties, the proportion of links in the first and third quadrant grows rapidly to ∼89.2% (2887 links) when λ=0.025 and then remains stable at ∼98.5% when λ is larger than 0.125. These results suggest the high consistency between the traditional correlation-based networks and our proposed LRML network.

**Figure 4:**
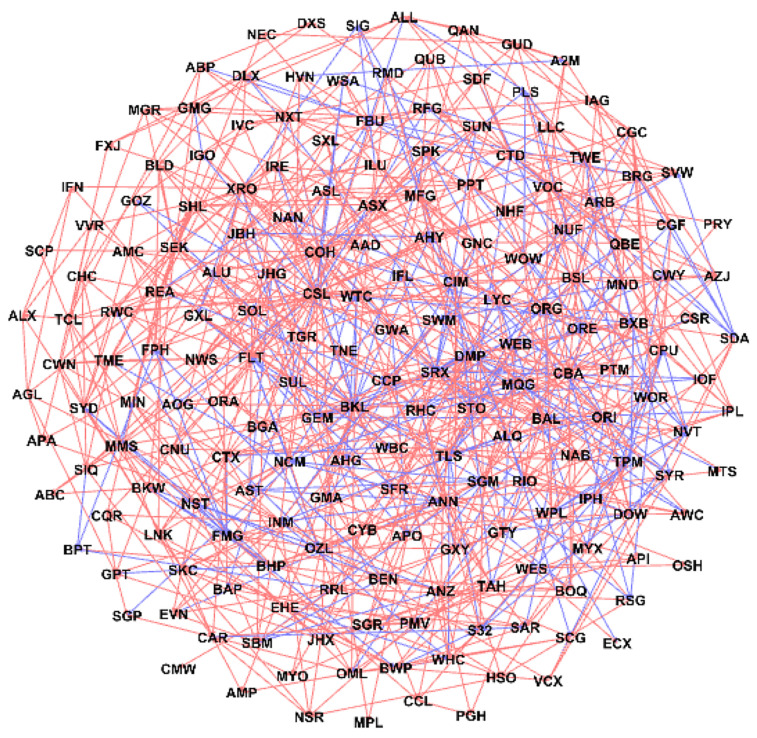
The ASX 200 stock network generated by the proposed linear relationship model with LASSO using penalty factor λ=0.189. Red and blue lines represent positive and negative links in the generated network respectively.

Figure 4 presents the stock relationship network generated by our LRML model with λ=0.189. This penalty value is chosen so that the number of directed links in this developed network is twice the number of edges in the PMFG network (i.e., 3N−6=576). These 1152 directed edges consist of 908 positive and 244 negative ones, and 261 and 630 links are bilateral and unilateral, respectively. Based on the results in Figure 3C, the positive and negative relations shown in Figure 4 are highly consistent with the Pearson correlations.

### 3.4. Consistency of MST and LRML Networks

We next examine whether the LRML networks have a similar structure as networks derived from the previous approaches. This work studies the proportion of mutual links appearing in both MST and LRML networks. The MST algorithm reduces the complete network to a minimum connected structure but still maintains the hierarchical clustering of the stocks. Other methods, such as the asset graph and the PMFG, primarily or even entirely remain the structure of MST [13,33,34]. Here we discuss two types of MST networks. The first one only considers positive correlations by using distance measure $d_{ij}=\sqrt{2(1-\rho_{ij})}$, and the second one includes both positive and negative correlations by using distance $d_{ij}=\sqrt{2(1-|\rho_{ij}|)}$, where $\rho_{ij}$ is the correlation coefficient for the prices of stocks *i* and *j*.

**Figure 5:**
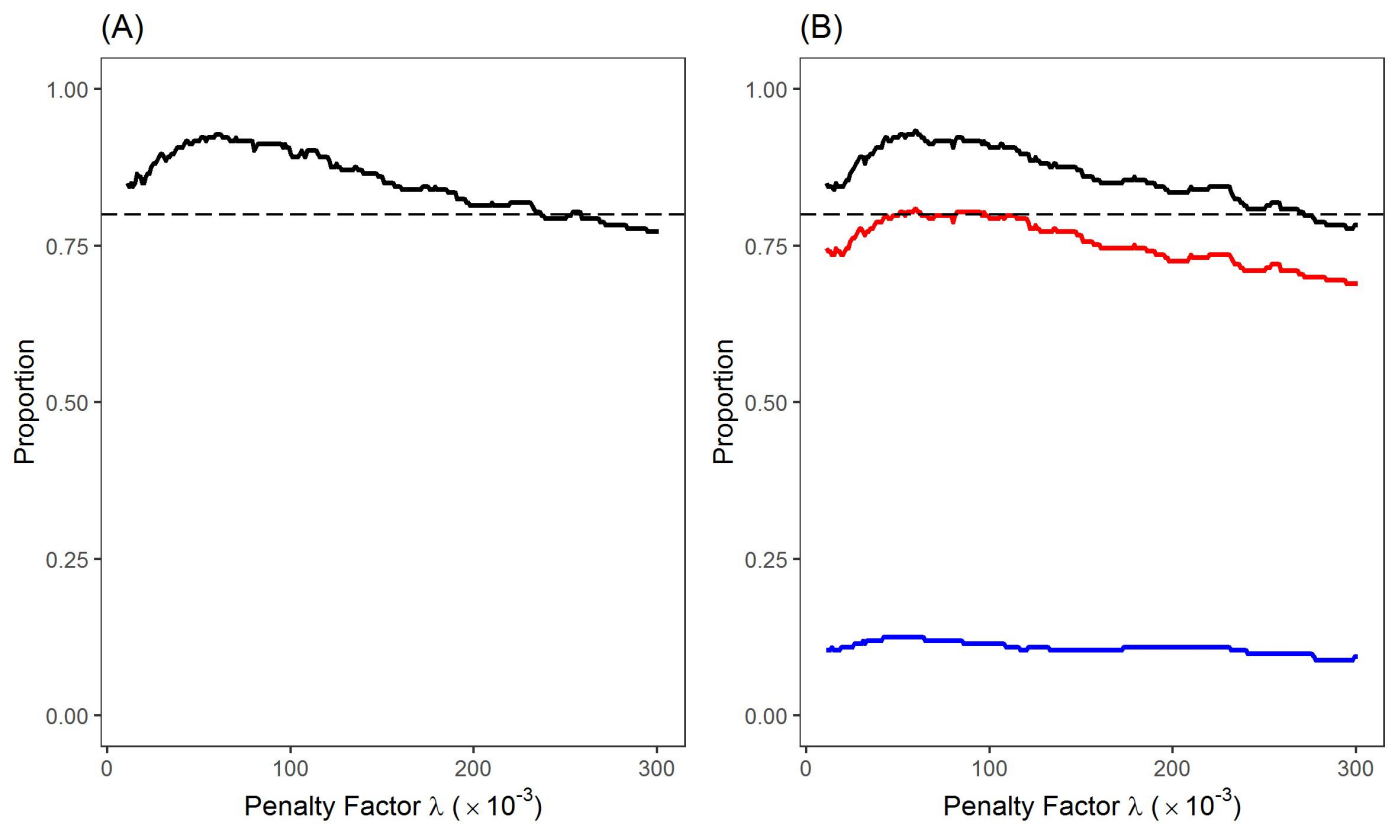
The proportions of mutual links between the MST Networks with the proposed LRML networks. (**A**). Proportions of mutual links in the first MST network with positive correlations only (Dash-line: y=0.8). (**B**). Proportions of mutual links with the second MST network with both positive and negative correlations (Dash-line: y=0.8; Black solid-line: proportions of all links; Red solid-line: proportions of positive links; Blue solid-line: proportions of negative links).

Figure 5 shows the proportions of the links appearing in the MST networks that are also in our proposed LRML networks determined by different λ values. A small value of the penalty factor (λ<0.01) leads to LRML networks with nearly all directed links and increases the likelihood of heterogeneous links. Here a heterogeneous link means that two edges for a node pair have the opposite signs. Thus, we provide the results in Figure 5 only for relatively large penalty factors (i.e., λ>0.01). In fact, the proportion values in Figure 5 increase gradually when λ rises from zero, and then reach the peak values when λ∼=0.059.

Figure 5A gives the proportions of the mutual links with the first network with positive correlations only, implying that our proposed model maintains the main structure of the MST network. When λ=0.059, the number of mutual links in the first MST and our networks reaches the maximum value of 179 links, accounting for 92.7% of the MST links. For the network in Figure 4 that is determined by λ=0.189, our model shares 161 links with the first MST network which accounts for 83.4% of the total MST links. When the penalty factor is greater than 0.059, there is a slight downward trend in the proportions of mutual links, which results from the sparser structure of LRML networks. Figure 5B presents similar observations for the proportions of the shared links between our network with the second network involving both positive and negative correlations. The total shared links with the second MST network are slightly higher than those with the first MST network because negative relations are considered in the second MST network. This result suggests again that our proposed network maintains the main hierarchical structure of the stock market. However, since the number of links in an MST network is fixed, the addition of certain negative links means the removal of other positive links.

### 3.5. Topological Properties of the LRML Network

**Figure 6:**
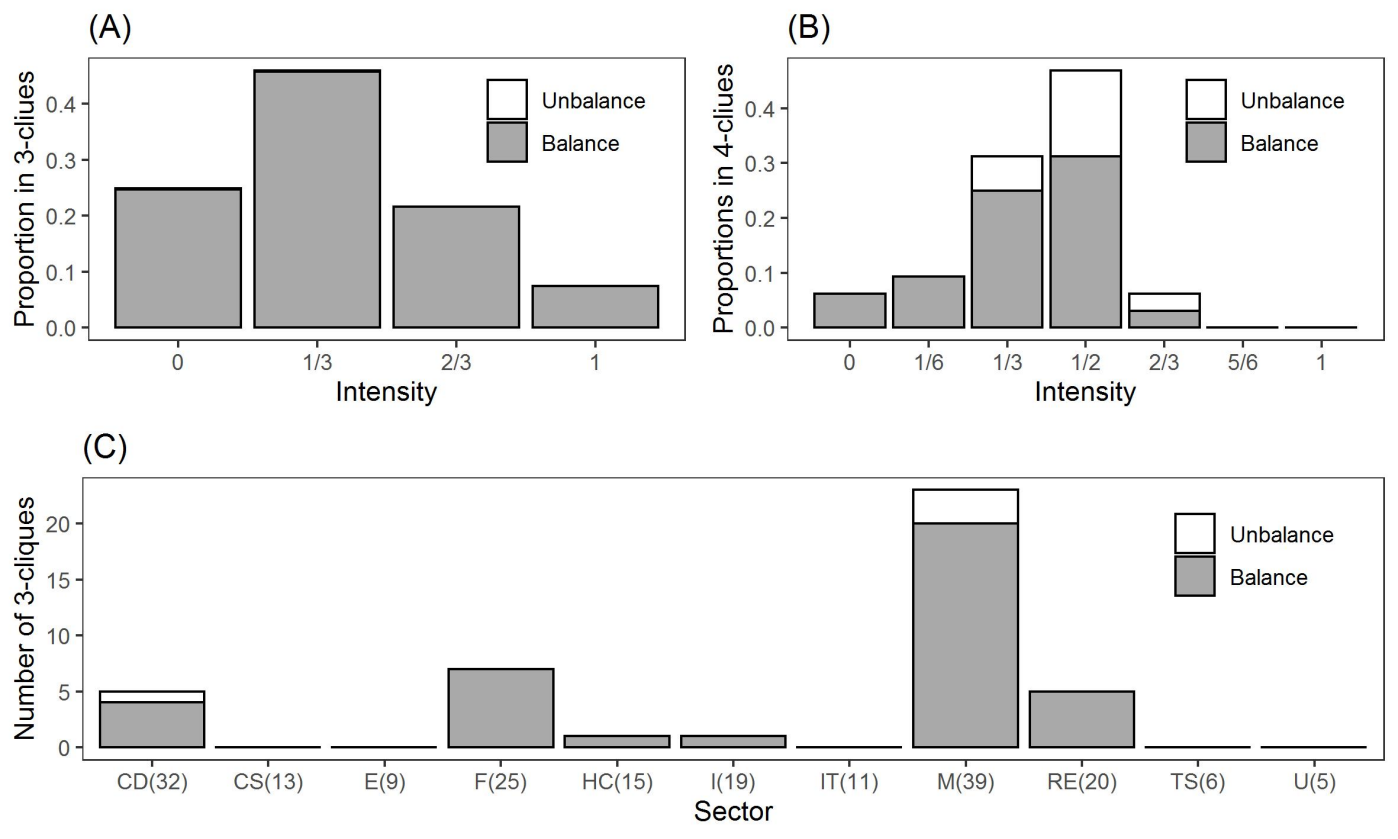
Intensity and balance properties of cliques. (**A**). Proportions in 3-cliques with different intensities. (**B**). Proportions in 4-cliques with different intensities. (**C**). Numbers of the 457 3-Cliques in 11 sectors (CD: Consumer Discretionary, CS: Consumer Staples, E: Energy, F: Finance, HC: Health Care, I: Industrials, IT: Information Technology, M: Materials, RE: Real Estate, TS: Telecommunication Service, U: Utilities. Number in brackets is the stock number of each sector).

Next we investigate the topological properties of the LRML network in Figure 4. The intensity of a clique indicates the ratio of the existing directed link number to the maximum link number in a given clique. For the 3-cliques in Figure 6A, about a quarter of 3-cliques have only one directed link for all the node pairs, namely with intensity 0. In addition, nearly a half of 3-cliques have one directed link for two node pairs and two directed links for one node pair, namely with intensity 1/3. However, the number of cliques with intensity 1 is quite low. Similar observations can be found for the intensities of the 4-cliques. In this case, nearly one-third of 4-cliques and a half of 4-cliques have intensities 1/3 and 1/2, respectively. None of the 4-cliques has 5 or 6 two-directed edges, namely with intensity 5/6 or 1. Overall, the majority of cliques in this network has low intensity values.

**Figure 7:**
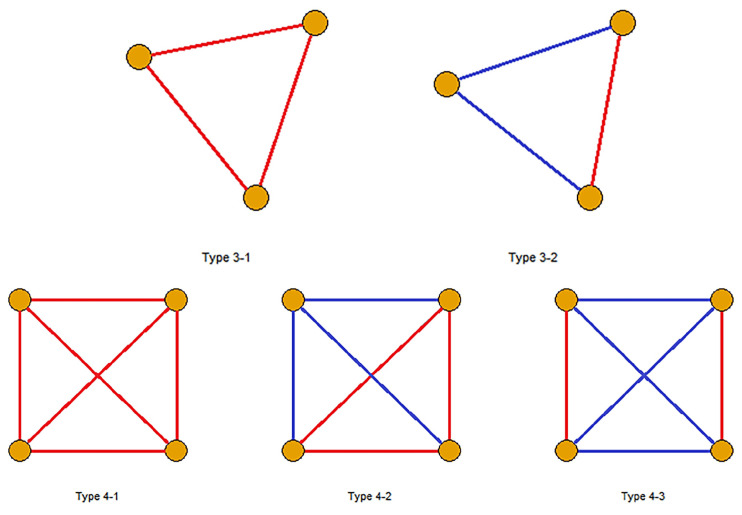
Types of 3-cliques and 4-cliques. Top: two types of 3-cliques. Bottom: three types of 4-cliques. (Red and blue lines represent positive and negative links respectively).

Regarding the balanced property, there are 455 balanced 3-cliques, which are out of the total 456 3-cliques as shown in Figure 6A. In particular, 306 of them are connected by three positive links (type 3-1 in Figure 7), implying strong relationships between these stocks. In addition, 149 of them have one positive link and two negative links (type 3-2), which corresponds to the fact that “enemies’ enemies are my friends”. In addition, Figure 6C shows the stocks in these 3-cliques distribute quite differently in 11 sectors. Among them, stocks in the materials sector account for more than a half of 3-cliques, followed by the stocks in finance, consumer discretionary and real estate sectors. These four sectors show stronger internal connections, while stocks in the other sectors are prone to develop external relations.

Although the structure of 4-cliques is more complex than that of 3-cliques, all of 32 4-cliques in the built network can be classified into three types of cliques in Figure 7. Type 4-1 cliques have four positive links and represent strong ties between stocks. However, both 4-2 and 4-3 types present a separate layout dividing the given 4-clique into two components so that positive edges exist within one part and negative edges connect in the other part. We also find that only two 4-cliques belong to the single sector (i.e., materials sector), implying that most nodes in the 4-cliques tend to develop connections outside their sectors.

## 4. Conclusions

In this work, we propose a new linear relationship model with LASSO (LRML) to describe the synchronous relationships between stocks. The penalty factor in LASSO determines the density of generated networks. Based on the existing correlation-based methods, our model extends the current approaches to study both positive and negative relations concurrently. Compared with existing methodologies dominantly using the paired relationship, the new method can describe the influence of all stock prices in a systemic way. As an application, we utilise the proposed model to develop the stock networks based on the ASX200 stock market data. Our results show that the proposed linear relationship model is highly consistent with correlation measurements in both positive and negative correlations, and the developed network maintains the main hierarchical structure of the stock market. Furthermore, we use the balance theory to investigate the topological properties of the stock network. This work suggests that the proposed method is a powerful tool to develop stock relationship networks by including negative correlations and also by investigating the influence of stock prices in a systemic way.

The directed links in our proposed model, together with the balanced theory, provide further insight into the topological properties of stock relationship networks. Our results demonstrate that stocks are highly prone to form balanced local connections, especially in triad relationships. Only two types of 3-cliques are found in the established network, and the existing types of 4-cliques are much fewer than all possible types, which implies that ASX 200 stocks may behave in specific particular patterns. In addition, strong local connections are more likely to exist between different sectors rather than in the same one. This work represents a further step in the study of directed links and balanced theory. Another important and interesting topic is the application of network theory to portfolio selection, which employs the financial market network as a useful approach to improve the portfolio selection process by targeting a group of assets according to their centrality [53,54,55]. One of the important topics is the comparison of the risk-return of new network methods with that obtained from Harry Markovic’s mean-variance model. All these interesting questions will be the topics of further research.
